# Fish occurrence in the Kama River Basin (Russia)

**DOI:** 10.3897/BDJ.10.e89169

**Published:** 2022-08-16

**Authors:** Ivan Pozdeev, Oleg Artaev, Sergei Ogorodov, Ilya Turbanov, Aleksey Bolotovskiy, Boris Levin

**Affiliations:** 1 Perm Branch Russian Federal "Research Institute of Fisheries and Oceanography" (VNIRO), Perm, Russia Perm Branch Russian Federal "Research Institute of Fisheries and Oceanography" (VNIRO) Perm Russia; 2 Perm State University, Perm, Russia Perm State University Perm Russia; 3 Papanin Institute for Biology of Inland Waters Russian Academy of Sciences, Borok, Yaroslavl' Prov., Russia Papanin Institute for Biology of Inland Waters Russian Academy of Sciences Borok, Yaroslavl' Prov. Russia; 4 Severtsov Institute of Ecology and Evolution Russian Academy of Sciences, Moscow, Russia Severtsov Institute of Ecology and Evolution Russian Academy of Sciences Moscow Russia; 5 Cherepovets State University, Cherepovets, Russia Cherepovets State University Cherepovets Russia

**Keywords:** freshwaters, fish fauna, occurrence, distribution, Kama, Volga

## Abstract

**Background:**

Dataset contains information on fish occurrences in the Kama River Basin (Russian Federation). The Kama River is the largest tributary (1805 km) of the Volga River and is geographically often considered the main river due to the larger volume of water at their confluence.

**New information:**

Dataset is based on our own field studies conducted during 2008-2021. It includes 6,447 occurrences relating to 48 taxa, 46 of which were identified at species level and two at the genus level. All occurrences have coordinates and belong to 13 families of Actinopterygii. All presented data are published for the first time.

## Introduction

Overall, the fish fauna of the Kama River system is similar to that of the Volga River, except for brackish water species from the estuary of the Volga. Indeed, the Kama Basin serves as home for the Volga endemic fish species like the Volga gudgeon *Gobiovolgensis*. In addition, the Kama water system is drained from the Ural Mountains with an extended mountain zone in the upper reach of tributaries and serves as a refuge for cold-water fish species (*Huchotaimen*, resident populations of *Salmocaspius*, *Thymallusthymallus*, *Cottuskoshewnikowi*), which are mostly impacted by human activity and habitat degradation. For example, the Volga population of *H.taimen* is thought to be extirpated in the upper Volga and only the Kama population survived (Hogan and Jensen 2012). Data on genetic diversity of fishes from the Kama are sporadic (Mendel et al. 2008, Marić et al. 2014, Levin et al. 2017, Segherloo et al. 2021). At the same time, our unpublished genetic data argue for presence of the unique Kama populations. Published literature data are scarce in relation of fish occurrences (Zinovjev et al. 2004, Bogdanov et al. 2006, Slynko and Tereshchenko 2014, Bezmaternykh and Shcherbina 2018, Mikheev and Ogorodov 2015, Kotelnikova 2016, Karabanov et al. 2018, Makhrov et al. 2020). Biodiversity data and fish occurrences data, in particular, are strongly needed information for performing qualitative research in aquatic ecosystems. The data on fish occurrences in the Kama Basin are important also for management of local fishery resources, as well as for more focused conservation efforts in relation to the rare or threatened species.

## General description

### Purpose

The purpose of this article is to make publicly available our data on fish occurrences in the Kama Basin. The placement of the dataset on the GBIF platform will facilitate further comprehensive studies on fish fauna.

### Additional information

Ichthyological observations in the Kama Basin began from 1918 by the establishment of a biological station by the Society of Naturalists at Perm University (Pidemskiy 2013). Large water bodies like Kamskoe and Votkinskoe Reservoirs were rather in focus of ichthyological studies during 20^th^ century, while small rivers, brooks and streams began to be scientifically explored only recently. Ichthyological surveys of small water bodies and streams significantly improve knowledge on local fauna, especially in terms of occurrence and distribution of rare and endangered species (Pozdeev et al. 2021). Localities in our dataset were selected to cover the Kama Basin, as well as various habitats most comprehensively.

The presented information on species occurrences may be used by ichthyologists, ecologists, conservation biologists and managers in the area of nature protection.

## Sampling methods

### Study extent

The dataset contains information on 6,447 occurrences for 48 taxa. The occurrences were recorded during the years 2008-2021. The study area is ~ 507,000 km².

### Sampling description

Fish were sampled using various fishing gear – gill nets and drift gill nets with mesh size from 10 to 100 mm, seine nets, frame nets, electrofisher ELLOR-2 (Russia, Saint-Petersburg) and fishing rod. The sampling was done accordingly with permissions of local authorities.

### Quality control

Each observation contains information on locality (coordinates), date, name of water bodies, name of observer and name of identifier. Geographical coordinates for sampling localities were detected using satellite navigation systems or using Google Maps and Yandex Maps services. Species identification was done, based mainly on the morphological characters or in combination with both morphology and DNA barcodes (COI) originally obtained by the authors. DNA barcodes were obtained according to a protocol following Ivanova et al. (2007). They were compared with DNA barcodes already placed to GenBank (www.ncbi.nlm.nih.gov) using service BLAST (optimised search for highly similar sequences with expected threshold of 0.05 and other settings were as default).

## Geographic coverage

### Description

**Figure 1.**
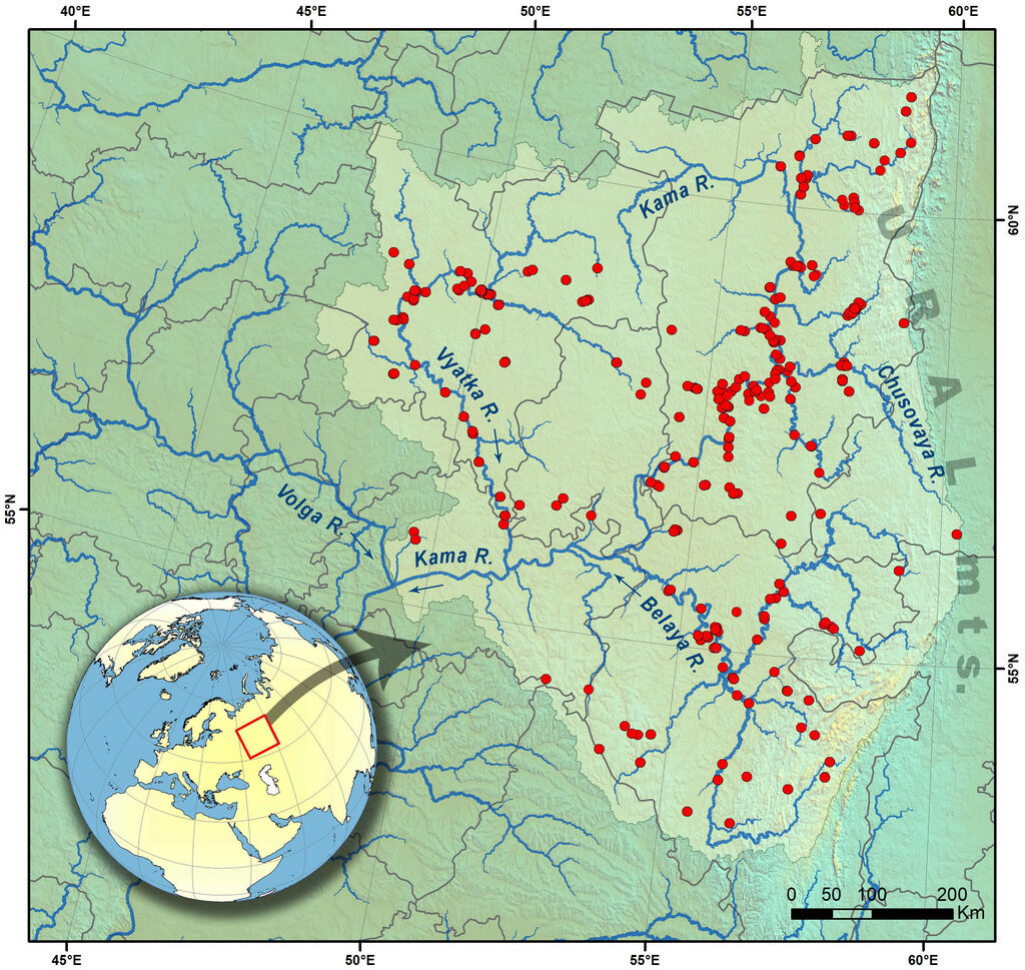
Map of sampling sites in the Kama River Basin. The map was created in ArcGIS 10.8 software (www.esri.com).

Kama Basin is located at the eastern part of the East European Plain; the most eastern tributaries drained from the western slope of the Ural Mountains. The Kama system covers an area ca. 1000 km from north to south and ca. 800 km from west to east. The length of the Kama River is 1805 km and the area of the Basin is ca. 507,000 km². The largest tributaries of the Kama are the Belaya R. (1430 km), Vyatka R. (1314 km), Chusovaya R. (592 km) and Vishera R. (415 km) (Fig. 1). The basin of the Kama River is characterised by various terrestrial and riverine landscapes and habitats ranging from plain to highlands. Notably, the Kama Basin has been connected with the Arctic Ocean drainage via the upper reaches of the Southern Kel’tma (Caspian Sea Basin) and the Northern Kel’tma (White Sea Basin) rivers in the past (Nazarov et al. 2019, Nazarov et al. 2020). Starting from 1822, these rivers were interconnected for 20 years via the Northern Ekaterininsky canal (Klimenko 2011). The Kama Basin could have a connection with the Arctic Ocean catchment also via the Chusovaya River, whose source in Siberia is surrounded by the Ob’ Basin.

### Coordinates

52.7° and 61.9° Latitude; 47.2° and 60.4° Longitude.

## Taxonomic coverage

### Description

The dataset includes 48 taxa, of which 46 were identified at species level (one species with inaccurate identification) and two at generic level (Table 1). Taxonomy is given according to Fricke et al. (2021).

During the 20^th^ century, the fish fauna of Kama Basin was significantly re-arranged. Species diversity of the anadromous species (fam. Petromyzontidae, Acipenseridae, Salmonidae) has been significantly lowered due to the construction of numerous dams on the Volga River. At the same time, some exotic and invasive species have been recorded. The most numerous populations of the alien species were established by *Clupeonellacultriventris*, *Perccottusglenii* and *Neogobiusmelanostomus*. Apart from naturalised alien species, aquaculture species like *Ctenopharyngodonidella*, *Hypophthalmichthysmolitrix* and *Oncorhynchusmykiss* are being occasionally recorded during the 20^th^-21^st^ centuries.

The dataset contains two species of the genus *Rutilus* – *R.rutilus* and *R.lacustris* according to a recent genetic study (Levin et al. 2017). Their occurrences are given, based on the genetic data of Levin et al. (2017) and Artaev et al. (2021) since species identification by morphology in the zone of their sympatry has not yet been developed. Occurrences of *Rutilus* without genetic confirmation were referred to R.cf.lacustris, based on its major predominance in the Kama Basin (Artaev et al. 2021). We also consider the Prussian carp *Carassius ‘gibelio*’ as a *C.auratus* species complex because its taxonomic status is still under debate (Wouters et al. 2012, Rylková et al. 2013, Vekhov 2013, Šimková et al. 2015).

## Temporal coverage

**Data range:** 2008-8-15 – 2021-3-30.

### Notes

Only our own data are included. The period of observation is from 2008 to 2021. The significant portion of observations (ca. 40%) was done during May, a period of spring flooding and massive spawning migrations. Observations have been also performed during other months, except for January and February.

## Usage licence

### Usage licence

Creative Commons Public Domain Waiver (CC-Zero)

## Data resources

### Data package title

Fish occurrence in Kama River Basin

### Resource link

https://www.gbif.org/dataset/a96f7777-8222-4f17-be6d-295d8d067766

### Alternative identifiers

https://doi.org/10.15468/gea4r4

### Number of data sets

1

### Data set 1.

#### Data set name

Fish occurrence in Kama River Basin

#### Data format

DwC-A

#### Character set

UTF-8

**Data set 1.** 

Column label	Column description
occurrenceID	The Globally Unique Identifier number for the record.
basisOfRecord	The specific nature of the data record: HumanObservation.
eventDate	Date format as YYYY-MM-DD
scientificName	The full scientific name including the genus name and the lowest level of taxonomic rank with the authority.
kingdom	The full scientific name of the kingdom in which the taxon is classified.
phylum	The full scientific name of the phylum or division in which the taxon is classified.
class	The full scientific name of the class in which the taxon is classified.
order	The full scientific name of the order in which the taxon is classified.
family	The full scientific name of the family in which the taxon is classified.
decimalLatitude	The geographic latitude of location in decimal degrees.
decimalLongitude	The geographic longitude of location in decimal degrees.
Country	The name of the country (Russia).
countryCode	The standard code for the country in which the Location occurs.
individualCount	The number of individuals represented present at the time of the Occurrence.
year	Year of the event was recorded.
month	The month of the event was recorded.
day	The integer day of the month on which the Event occurred.
recordedBy	A person or group responsible for recording the original Occurrence.
identifiedBy	A list of names of people, who assigned the Taxon to the subject.
waterBody	The name of the water body in which the Location occurs.
coordinateUncertaintyInMetres	The horizontal distance (in metres) from the given decimalLatitude and decimalLongitude describing the smallest circle containing the whole of the Location.
geodeticDatum	The ellipsoid, geodetic datum or spatial reference system (SRS) upon which the geographic coordinates given in decimalLatitude and decimalLongitude are based.
associatedReferences	Bibliographic reference of literature associated with the Occurrence.
identificationQualifier	A brief phrase or a standard term ("cf.", "aff.") to express the determiner's doubts about the Identification.

## Figures and Tables

**Table 1.** Occurrences of fish taxa in the Kama Basin represented in the dataset.

**Scientific name**	**Number of occurrences**
Acipenseridae	
*Acipenserruthenus* Linnaeus, 1758	141
Clupeidae	
*Clupeonellacultriventris* (Nordmann, 1840)	11
Cobitidae	
*Cobitis* Linnaeus, 1758	9
*Cobitismelanoleuca* Nichols, 1925	1
*Cobitistaenia* Linnaeus, 1758	18
*Misgurnusfossilis* (Linnaeus, 1758)	3
Cottidae	
*Cottuskoshewnikowi* Gratzianov, 1907	25
Cyprinidae	
*Abramisbrama* (Linnaeus, 1758)	889
*Alburnoidesrossicus* Berg, 1924	17
*Alburnusalburnus* (Linnaeus, 1758)	181
*Ballerusballerus* (Linnaeus, 1758)	165
*Ballerussapa* (Pallas, 1814)	175
*Bliccabjoerkna* (Linnaeus, 1758)	521
*Carassiusauratus* (Linnaeus, 1758) species complex	261
*Carassiuscarassius* (Linnaeus, 1758)	39
*Chondrostomavariabile* Yakovlev, 1870	32
*Ctenopharyngodonidella* (Valenciennes, 1844)	8
*Cyprinuscarpio* Linnaeus, 1758	21
*Gobiovolgensis* Vasil'eva, Mendel, Vasil'ev, Lusk & Luskova, 2008	102
*Hypophthalmichthysmolitrix* (Valenciennes, 1844)	5
*Leucaspiusdelineatus* (Heckel, 1843)	2
*Leuciscusaspius* (Linnaeus, 1758)	157
*Leuciscusidus* (Linnaeus, 1758)	359
*Leuciscusleuciscus* (Linnaeus, 1758)	176
*Pelecuscultratus* (Linnaeus, 1758)	258
*Phoxinus* Rafinesque, 1820	107
*Rhynchocyprispercnurus* (Pallas, 1814)	1
*Romanogobioalbipinnatus* (Lukasch, 1933)	4
*Rutiluslacustris* (Pallas 1814)	14
Rutiluscf.lacustris (Pallas 1814)	902
*Rutilusrutilus* (Linnaeus, 1758)	7
*Scardiniuserythrophthalmus* (Linnaeus, 1758)	124
*Squaliuscephalus* (Linnaeus, 1758)	166
*Tincatinca* (Linnaeus, 1758)	66
Esocidae	
*Esoxlucius* Linnaeus, 1758	365
Gobiidae	
*Neogobiusmelanostomus* (Pallas, 1814)	2
Lotidae	
*Lotalota* (Linnaeus, 1758)	75
Nemacheilidae	
*Barbatulabarbatula* (Linnaeus, 1758)	82
Odontobutidae	
*Perccottusglenii* Dybowski, 1877	12
Percidae	
*Gymnocephaluscernua* (Linnaeus, 1758)	197
*Percafluviatilis* Linnaeus, 1758	896
*Sanderlucioperca* (Linnaeus, 1758)	498
*Sandervolgensis* (Gmelin, 1789)	9
Salmonidae	
*Coregonusmuksun* (Pallas, 1814)	1
*Huchotaimen* (Pallas, 1773)	8
*Oncorhynchusmykiss* (Walbaum, 1792)	2
*Salmocaspius* Kessler, 1877	6
*Thymallusthymallus* (Linnaeus, 1758)	60
Siluridae	
*Silurusglanis* Linnaeus, 1758	57
